# Enhanced Sulfur Transformation by Multifunctional FeS_2_/FeS/S Composites for High‐Volumetric Capacity Cathodes in Lithium–Sulfur Batteries

**DOI:** 10.1002/advs.201800815

**Published:** 2019-02-10

**Authors:** Kai Xi, Deqing He, Chris Harris, Yuankun Wang, Chao Lai, Huanglong Li, Paul R. Coxon, Shujiang Ding, Chao Wang, Ramachandran Vasant Kumar

**Affiliations:** ^1^ Department of Materials Science and Metallurgy University of Cambridge Cambridge CB3 0FS UK; ^2^ School of Chemistry and Materials Chemistry Jiangsu Normal University Xuzhou Jiangsu 221116 P. R. China; ^3^ Department of Applied Chemistry School of Science MOE Key Laboratory for Nonequilibrium Synthesis and Modulation of Condensed Matter State Key Laboratory for Mechanical Behavior of Materials State Key Laboratory of Electrical Insulation and Power Equipment Xi'an Jiaotong University Xi'an 710049 P. R. China; ^4^ Department of Precision Instrument Tsinghua University Beijing 100084 China

**Keywords:** catalytic effect, density functional theory (DFT) calculations, FeS_2_/FeS/S composites, lithium–sulfur batteries, volumetric energy density

## Abstract

Lithium–sulfur batteries are currently being explored as promising advanced energy storage systems due to the high theoretical specific capacity of sulfur. However, achieving a scalable synthesis for the sulfur electrode material whilst maintaining a high volumetric energy density remains a serious challenge. Here, a continuous ball‐milling route is devised for synthesizing multifunctional FeS_2_/FeS/S composites for use as high tap density electrodes. These composites demonstrate a maximum reversible capacity of 1044.7 mAh g^−1^ and a peak volumetric capacity of 2131.1 Ah L^−1^ after 30 cycles. The binding direction is also considered here for the first time between dissolved lithium polysulfides (LiPSs) and host materials (FeS_2_ and FeS in this work) as determined by density functional theory calculations. It is concluded that if only one lithium atom of the polysulfide bonds with the sulfur atoms of FeS_2_ or FeS, then any chemical interaction between these species is weak or negligible. In addition, FeS_2_ is shown to have a strong catalytic effect on the reduction reactions of LiPSs. This work demonstrates the limitations of a strategy based on chemical interactions to improve cycling stability and offers new insights into the development of high tap density and high‐performance sulfur‐based electrodes.

## 1 Introduction

The development of portable electronic devices, electric vehicles, and large‐scale energy storage has spurred extensive research toward batteries with higher energy densities, lower costs, and longer cycle life.^1^, ^2^ Among the alternatives for conventional lithium‐ions batteries, the lithium–sulfur (Li–S) battery is an energy storage system of particular interest, due to its high theoretical energy density of up to 2567 Wh kg^−1^.^3^, ^4^, ^5^, ^6^, ^7^ Combining the advantages of relative abundance, low cost, and the environmental friendliness of sulfur, Li–S batteries have been considered as one of the most promising candidates for the next‐generation of batteries.^1^, ^2^, ^3^, ^4^, ^5^, ^6^, ^7^ However, any practical applications of Li–S cells are hindered by several significant drawbacks, including the sluggish and unstable electrochemical kinetics of redox chemistry proceeds of sulfur, lack of a scalable synthesis of the electrode composite materials, and the low volumetric energy density of the sulfur‐based composite.^3^, ^4^, ^5^, ^6^, ^7^

The unfavorable electrochemical kinetics of sulfur are caused by a combination of factors, such as the insulating nature of element sulfur and its reduction products, Li_2_S_2_ and Li_2_S; the dissolution/diffusion of polysulfides; and the substantial volume expansion occurring (up to 80%) upon discharge.^3^, ^4^, ^5^, ^6^, ^7^ As such, two main strategies have been developed for constructing composite cathode materials that can suppress these issues. The first method introduces a conductive host into the sulfur electrode to increase conductivity, provide void space to buffer against volume fluctuations, and physically confine the dissolution of the polysulfide by means of a nanoporous structure.^3^, ^4^, ^5^, ^6^, ^7^, ^8^, ^9^, ^10^, ^11^, ^12^, ^13^, ^14^ Various materials, such as microporous carbon,^8^, ^9^ mesoporous carbon,^10^ hierarchical porous carbon,^11^, ^12^ carbon nanotubes,^13^ graphene, and their hybrids,^14^, ^15^, ^16^, ^17^ have been used as supporting materials for sulfur, and a significantly enhanced electrochemical performance has been obtained for Li–S cells using this design. However, conjugate nonpolar carbon materials interact weakly with polar lithium polysulfides, resulting in reduced cycle life. This is because polysulfides gradually diffuse into the electrolyte from the carbon surface.^18^ Consequently, another strategy based upon strong polar–polar interactions could be more attractive. Various polar compounds, such as heteroatom doped carbon,^15^, ^16^ metal oxides,^19^ metal sulfides,^20^, ^21^ and MOFs,^22^, ^23^ have been investigated as sulfur host materials. This route is based on the strength of interfacial chemical interactions rather than spatial confinement, and a significantly improved long‐term cycling performance is obtained.^18^, ^24^ The strong polar–polar interactions based on interfacial phenomena are highly dependent upon the direct contact area between the host material and the polysulfide.^19^, ^20^, ^21^, ^22^, ^23^ However, it is difficult to achieve a completely uniform dispersion owing the poor affinity between nonpolar sulfur and polar compounds, even using a solution method. Meanwhile, most of the research has been focused on the selection of polar compounds and the fabrication of composites; while there have been comparatively few studies on their restricting mechanisms, for example, such as the binding direction of polysulfide on the surface of host materials.^18^, ^20^, ^24^, ^25^

In addition to the choice of sulfur host material, there are several other important parameters that must be optimized for the assembly of practical Li–S batteries, such as tap density, material consistency, and cost of the sulfur based electrode.^26^, ^27^, ^28^, ^29^ The volumetric energy density of batteries is highly dependent on the tap density of electrode materials.^26^, ^27^ For the same mass loading, a low tap density can cause a low volumetric capacity, thicker electrodes, and a longer electron pathway, thus yielding poor electrochemical performance.^30^ Unfortunately, elemental sulfur only has a tap density around 0.8 g cm^−3^, which is much lower than that of commercial LiCoO_2_ (2.0–2.4 g cm^−3^) in lithium ion batteries,^31^, ^32^ and is a significant drawback in its potential use as a cathode in practical energy storage applications. In addition, after construction with various porous host materials (usually >30 wt% in the cathode), such as nanoporous carbon and nanosturcted beyond‐carbon materials, an inferior packing of particles will further effect the tap density of the sulfur‐based composite.^32^ At the same time, for the sake of material consistency, it is necessary to develop a large‐scale route for the synthesis of sulfur‐based composites with excellent electrochemical performance.^28^, ^29^ Thus, the challenge of developing a scalable route to synthesize sulfur‐based composites with both high tap density and satisfactory electrochemical performance will be an extremely important milestone for the further practical application of Li–S batteries.

**Figure 1:**
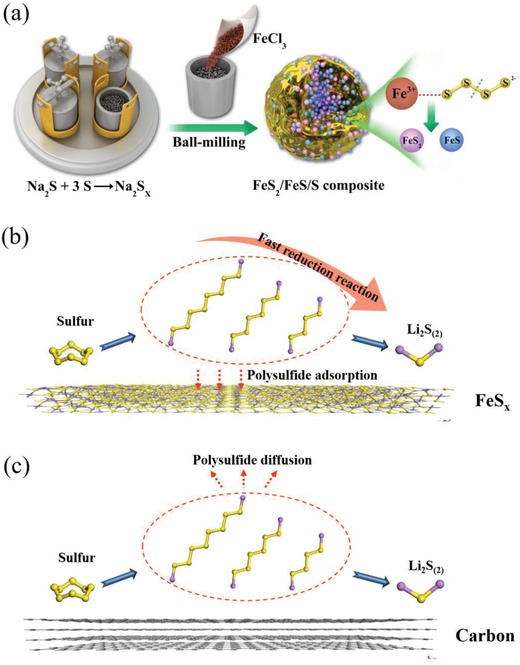
a) Schematic representation of the synthesis of FeS_2_/FeS/S composites and b,c) comparisons of the adsorption mechanisms of polysulfides onto the surface of different substrates.

To address these issues, we employed a redox reaction between Fe^3+^ and S*_x_* ^2−^ to generate a FeS_2_/FeS/S nanocomposite in situ, conducted by means of a facile continuous ball‐milling route, as shown in **Figure** **1**a. Such a design has many merits, including the provision of a sufficiently high contact area between sulfur and FeS*_x_* to adequately restrict polysulfide dissolution, the potential for scalable synthesis to ensure material consistency, and an ordered and dense packing of the various components to produce a high tap density. Iron (Fe^3+^ in this work), the most common element by mass on earth,^33^ was chosen as the precursor, as generated FeS and FeS_2_ are expected adsorb polysulfides and accelerate the cleavage of long‐chain polysulfides, more effectively than carbon materials as shown in Figure 1b,c. In addition, FeS is an excellent electronic conductor (10–1000 S cm^−1^), and electroactive sulfides can also confer additional capacity to the composite.^25^, ^34^, ^35^ Naturally, a high discharge capacity and stable cycle performance can be obtained for the composites prepared here, and restricting mechanisms that occur are systemic presented via first‐principle calculations based on density functional theory (DFT). Moreover, the detailed interfacial catalytic mechanism was systematically investigated by electrochemical analysis, spectroscopic, and calculations.

## 2 Results and Discussion

**Figure 2:**
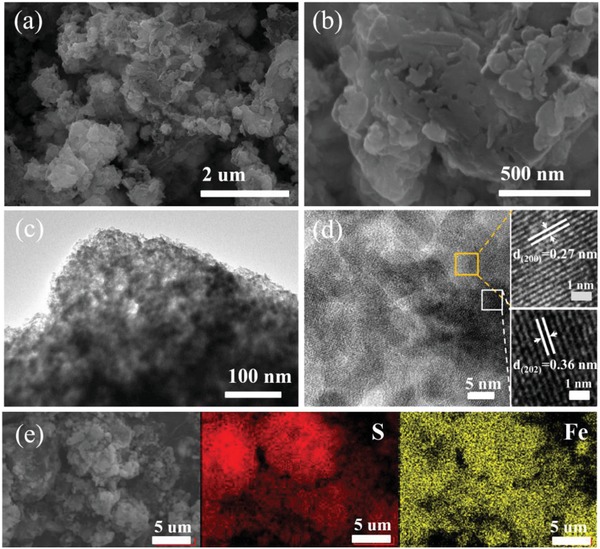
a,b) SEM and c,d) TEM images, along with elemental mapping of the FeS_2_/FeS/S composite.

The scanning electron microscopy (SEM) and transmission electron microscopy (TEM) images shown in **Figure** **2** illustrate the structural and morphology of the composite samples. As observed in Figure 2a, the FeS_2_/FeS/S composite consists of microsized particles with a large number of scattered nanoparticles. With an increase in magnification (Figure 2b), a relatively smooth surface can be seen and after washing away by an excess of chloroform (Figure S1a,b, Supporting Information), aggregates of nanoparticles are observed, which can be attributed as FeS_2_ and FeS nanoparticles. For the scattered nanoparticles, these may be derived from the washing process, which are contrasted with the unwashed ball‐milled samples presented in Figure S1c,d in the Supporting Information. The hierarchical inner structure is revealed in greater detail in the TEM images. At the edge of the particles, it is observed that FeSx nanoparticles interconnect to form a loose structure after sulfur evaporation as shown in Figure 2c. A high‐resolution TEM image is given in Figure 2d and a well‐resolved crystalline structure of sulfur and FeS_2_ are both detected, from which the lattice fringes of 0.27 and 0.36 nm can be assigned to the (200) plane of FeS_2_ and the (202) plane of sulfur, respectively.^36^ In addition, a distinct grain boundary between sulfur and FeSx is observed, indicating that sulfur and FeS*_x_* are tightly bound to some extent. Figure 2e shows the element mapping of the FeS_2_/FeS/S composite, from which it is apparent that the ferrous and sulfur are well inter‐dispersed. Based on above observations, it is can be concluded that FeS*_x_* nanoparticles are tightly bound by elemental sulfur to form a homogeneous composite.

**Figure 3:**
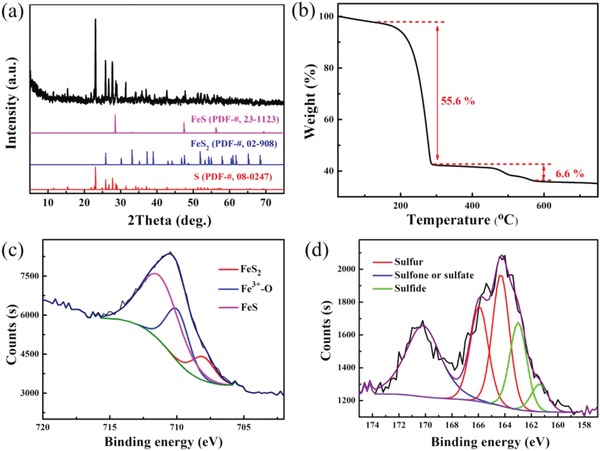
a) XRD patterns, b) TGA curve, and c) Fe 2p XPS spectra and d) S 2p XPS spectra of the FeS_2_/FeS/S composite.

The redox reaction between Fe^3+^ and S*_x_* ^2−^ can generate in situ FeS_2_, FeS, and sulfur in the composite,^37^ and thus the content of active sulfur in the composite is highly dependent on the value of “*x*.” However, when the molar ratio between sulfur and Na_2_S is above 3, it is difficult to obtain homogeneous NaS*_x_* without incurring significant levels of sulfur impurities during ball‐milling. Therefore, we synthesized the FeS_2_/FeS/S composite using a S: Na_2_S molar ratio of 3:1 in this work. **Figure** **3**a shows the X‐ray powder diffraction (XRD) spectra obtained for this composite, with the reflection peaks of FeS_2_, FeS, and S all being well detected. Thermogravimetric analysis (TGA) curves obtained from the FeS_2_/FeS/S composite, shown in Figure 3b, reveal a weight loss process that occurs in two stages. The first‐stage, occurring at temperatures of below 300 °C, can be attributed to the loss of sulfur,^15^ while the second‐stage, occurring at temperatures 400–600 °C, is correlated with the degradation of FeS_2_ to generate FeS and S.^37^, ^38^ At the end of the TGA measurement, there is ≈35.3 wt% FeS remaining. And the content of FeS_2_ and FeS in the composite can be calculated as 24.2 and 17.2 wt%, respectively. To further investigate the composition of the FeS_2_/FeS/S composite, XPS testing was performed. The Fe 2p X‐ray photoelectron spectroscopy (XPS) spectrum in Figure 3c shows three major peaks at 711.3, 710.0, and 708.0 eV, which can be attributed to FeS, Fe^3+^‐O, and FeS_2_, respectively.^34^, ^37^ On exposure to air, the black color of the FeS*_x_*/S composite gradually turns to brown (Figure S2, Supporting Information) and eventually combusts and thus the detection of Fe^3+^‐O may result from the oxidation of FeS*_x_* during the testing process. Figure 3d shows the S 2p spectra, and five peaks can be distinguished at 170.1, 165.9, 164.2, 163.0, and 161.5 eV, which correspond to sulfate, sulfur, and sulfide species, respectively.^8^, ^34^, ^37^, ^39^, ^40^ It should be emphasized that the binding energy of the S_2p3/2_ peak (163.1 eV) is lower than that of elemental sulfur (164.0 eV), indicating strong interactions between FeS*_x_* and sulfur.

**Figure 4:**
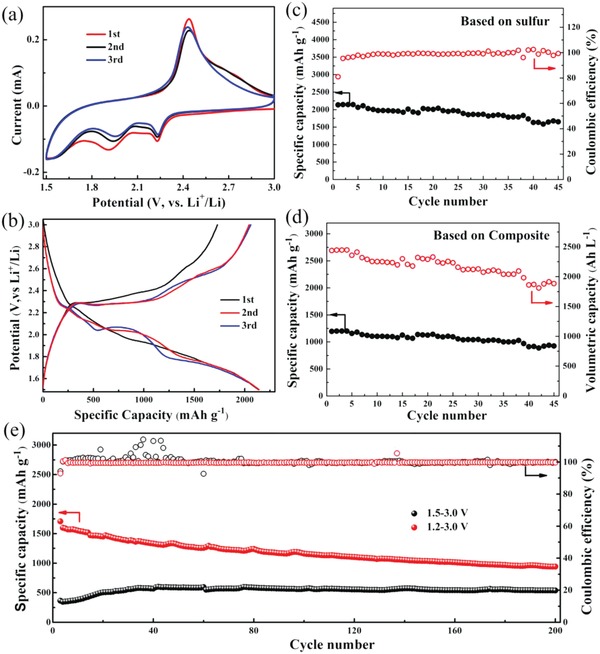
a) Cyclic voltammograms at a scan rate of 0.1 mV s^−1^, b) discharge and charge curves, c,d) cycling performance at a current density of 160 mA g^−1^ and e) 1600 mA g^−1^ of FeS_2_/FeS/S composites after an initial two cycles at a current of 160 mA g^−1^.

Cyclic voltammetry (CV) was used to evaluate the electrochemical behavior of the FeS_2_/FeS/S composite, as shown in **Figure** **4**a. Two typical cathodic peaks appearing at 2.23 and 2.0 V can be observed, which can be attributed to the conversion of sulfur to high‐order polysulfides and further to low‐order polysulfide (Li_2_S_2_ and Li_2_S), respectively.^20^, ^25^, ^29^, ^41^ In contrast to a conventional sulfur cathode, there are an additional broad peak appearing below 1.80 V. The lower sloping peak can be attributed to the reduction of FeS_2_, FeS, and polysulfides.^37^, ^42^, ^43^ When the voltage sweep is reversed the CV profile has a sharp peak at 2.44 V. The redox peaks appear relatively stable for the initial three cycles, indicating good reversibility in the composite cathodes. Figure 4b shows the initial three discharge–charge curves of the FeS_2_/FeS/S composite. The discharge profiles of the sample contain three major plateaus, which is consistent with the CV curves. Two plateaus were observed at ≈2.3 and 2.0 V, corresponding to the reduction process of sulfur to long‐chain soluble lithium polysulfides and short‐chain solid polysulfides, respectively.^20^, ^25^, ^29^, ^41^ In contrast to the conventional sulfur/carbon composite, an additional sloping plateau appears below 1.8 V, particularly in the second and third cycles. The capacity contribution from this sloping plateau reaches up to 911.0 mAh g^−1^ in the third cycle. This feature can be attributed to the synergistic effects of both the strong chemical adsorption of FeS*_x_* for polysulfides,^44^ and the additional lithium storage created from the conversion reaction of FeS*_x_*.^21^, ^34^, ^37^, ^42^ This could be confirmed by the detection of Li_2_FeS_2_ or Li_2−_ *_x_*FeS_2_ after cycling in Figure S3 in the Supporting Information. As a result, higher initial discharge and charge capacities of 2134.9 and 1728.8 mAh g^−1^, respectively, are obtained. This exceeds the theoretical capacity of elemental sulfur and is well retained after the second cycle. During subsequent cycling processes, the extra energy storage can be attributed to the reversible transformation between FeS_2_ and Li_2_FeS_2_.^21^, ^34^, ^37^, ^42^ It should be noted that the further reduction of Li_2_FeS_2_ to Fe and Li_2_S is unlikely to occur above 1.2 V (Figure S4a, Supporting Information).

The excellent cycle stability of FeS_2_/FeS/S composite can be better illustrated in Figure 4c. These show the discharge capacity to be well maintained, with a high coulombic efficiency of around 100%. After 30 cycles, a discharge capacity of 1865.5 mAh g^−1^ can still be achieved at the current density of 160 mA g^−1^. Taking into account the total mass of the composite, after 30 cycles, this equates to a capacity of 1044.7 mAh g^−1^ (Figure 4d). It should be highlighted that the FeS_2_/FeS/S composites exhibit a peak tap density of 1.4 g cm^−3^, which could translate to a compaction density of 2.04 g cm^−3^ and a volumetric capacity of 2031.1 Ah L^−1^ after 30 cycles (Figure 4d). Table S1 in the Supporting Information shows the volumetric capacity of FeS_2_/FeS/S composites based on these electrodes is much higher as compared to previous reports, further revealing its advantages for volumetric energy density. High cycle stability and discharge capacity also are obtained at the current density of 300 mA g^−1^, as shown in Figure S4b in the Supporting Information. When the cutoff potential is set at 1.0 V, an enhanced capacity is obtained, which results from the conversion reaction of Li_2_FeS_2_ to Fe and Li_2_S.^21^, ^34^, ^37^, ^42^ However, a much poorer cycle performance is presented due to significant structure changes within the composite (Figure S4b, Supporting Information). The cycling performance of FeS_2_/FeS/S composites at a high current density of 1600 mA g^−1^ is also given in Figure 4e. When the cutoff potential is set between 1.5 and 3.0 V, the composite demonstrates ultrastable cycle performance, but a low reversible capacity of 538.8 mAh g^−1^. This is due to extensive electrochemical polarization at this high level of current density. As such, a lower cutoff potential of 1.2 V can be used to obtain a much higher discharge capacity of 941.3 mAh g^−1^ after 200 cycles. This enhanced capacity can mainly be attributed to the increased utilization of active sulfur but not the reduction reaction of FeS and FeS_2_. The latter only occurs at potentials below 1.2 V and no potential plateau of FeS are observed after various cycles (Figure S5, Supporting Information). The high stability of FeS_2_/FeS/S composite electrodes can be further illustrated by the electrochemical impedance spectra (EIS) testing, and the Nyquist plots are given in Figure S6 in the Supporting Information. The charge transfer impedance associated with the semicircular arc in the high frequency range of the FeS_2_/FeS/S composite is well retained after the 1st (182.3 Ω) and 5th cycle (165.1 Ω), indicating that the structure of electrode is stable after several cycles.

**Figure 5:**
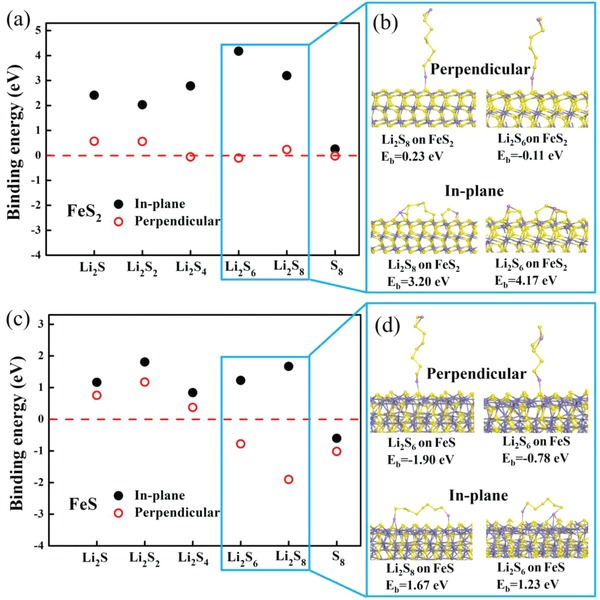
The formation energy between Li_2_S*_x_* (*x* = 1,2,4,6,8) and either a) FeS_2_ or b) FeS in different binding directions. Inset figures are optimized geometries of Li_2_S_6_ and Li_2_S_8_ on FeS_2_ and FeS surfaces.

As discussed above, we successfully prepared a uniform FeS_2_/FeS/S composite that exhibits both excellent cycle stability and high volumetric capacity. However, the detailed interaction mechanisms that occur within this composite remain unclear. In particular, the differences between the discharge–charge curves for this composite in comparison to those produced by conventional carbon/sulfur composites (Figure S7a, Supporting Information, shows an example of carbon nanotubes/sulfur (CNT/S) composites) are yet to be explained, and any restricting mechanisms caused by polysulfides are also poorly understood.^21^, ^34^ Therefore, first‐principle calculations based on DFT were performed in order to simulate the adsorption of both sulfur and Li_2_S*_x_* (*x* = 1, 2, 4, 6, 8) onto the surfaces of FeS_2_ and FeS. The results of this analysis are shown in **Figure** **5** and Figure S8 in the Supporting Information. Two configurations of adsorption are considered: one with the Li_2_S*_x_* chain lying in‐plane with the surface, and the other with the chain perpendicular to the surface. In‐plane adsorption is found to be more energetically favorable than perpendicular adsorption. For in‐plane adsorption, the calculated binding energies for adsorption onto FeS_2_ (Figure 5) are shown to be consistently larger than those calculated for adsorption onto FeS, indicating that FeS_2_ has stronger absorbability for lithium polysulfides than FeS does. Moreover, it is found that Li_2_S_6_ and Li_2_S_8_ tend to break down into two shorter chain segments when adsorbed onto FeS_2_. This implies that FeS_2_ has a strong catalytic effect on the cleavage reaction of high‐order polysulfides, which enables a fast reduction reaction during the discharge process. At the same time, it should be emphasized that FeS nanoparticles in the composite can also be partially oxidized by polysulfides to form FeS_2_.^34^ Thus, a sufficiently large number of catalytic and adsorbing sites in the form of FeS_2_ can lead to the generation of discharge curve that differ significantly from those produced by carbon/sulfur composites (Figure S7a, Supporting Information).

It should be noted that the dissolution of some polysulfides is unavoidable, and after long cycles in particular, this proportion will gradually increase. With regard to the adsorption of polysulfides in the solution; the question of the binding direction between polysulfides and host materials must not be overlooked. For example, it is apparent from the calculated binding energies that the sulfide matrix has weak or negligible absorbability for perpendicularly adsorbed polysulfides, with only one Li atom bonding with the surface sulfur atoms of FeS_2_ or FeS (Figure 5 and Figure S8, Supporting Information), and thus a decrease in capacity can be observed during long cycling of the composite (Figure 4c). Therefore, although polar compounds such as metal oxides and sulfides have been widely investigated as more suitable host materials for sulfur, they still do not present an ideal choice for obtaining an ultralong cycle life.

In addition to the strong chemical bonding illustrated by the experimental and calculation results, the affinity between FeS, FeS_2_, and the polysulfides can also impart a great influence on the kinetic performance of a Li–S battery, such its polarization and discharge capacity.^45^, ^46^, ^47^ We applied a symmetric cell approach to evaluate the interface transformation kinetics, using commercial conductive carbon paper (CP) as current collectors. A symmetric cell is composed of a Li_2_S_6_ electrolyte (2.5 m) between two identical CP‐based working electrodes loaded with or without FeS/FeS_2_ powders (0.45 mg cm^−2^). EIS of the Li_2_S_6_ symmetric cells are shown in Figure S9a in the Supporting Information. The semicircle in the high‐frequency region is related to the charge‐transfer process. The semicircle of FeS/FeS_2_‐CP exhibited a ≈57% decrease in diameter compared to that of pristine CP, which can be assigned to an enhanced charge transfer process in the FeS/FeS_2_ electrode. This decrease in *R*_ct_ (charge‐transfer resistance) should be attributed mainly to the improved interfacial affinity between FeS/FeS_2_ and polysulfides. In addition, we also carried out the CV tests to further illuminate the enhanced redox kinetics of LiPSs in the liquid phase. Figure S9b in the Supporting Information shows the polarization curves of Li_2_S_6_ symmetric cells within a voltage window of −0.8 to 0.8 V. The FeS/FeS_2_‐CP electrode exhibited a significantly larger redox current compared with that of CP, reflecting the promoted redox reaction of LiPSs in the liquid region and supporting the calculated results. The redox current results were in good accordance with the EIS data.

**Figure 6:**
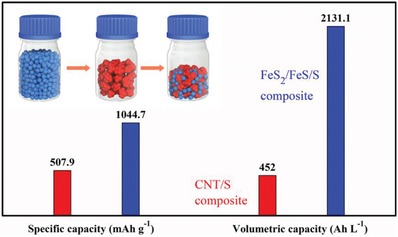
Comparisons of specific and volumetric capacity between FeS_2_/FeS/S composites and CNT/S composites. Inset is a schematic representation of the high tap density FeS_2_/FeS/S composite.

Tap density is another key property affecting the practical application of sulfur‐based composites, yet few reports focus on this parameter.^26^, ^27^, ^28^, ^29^, ^48^, ^49^ A schematic figure is provided which illustrates the origins of the high tap density found in our samples. As indicated in **Figure** **6**, the composites are microsized, with scattered nanoparticles, and generate denser stacking in comparison to either nanoparticles or micro–nano particles. To further illustrate the advantages of FeS_2_/FeS/S composites with such a high tap density, we can compare their specific capacity and volumetric capacity in relation to conventional carbon/sulfur composites (Figure 6 shown an example of CNT/S composites). As shown in Figure S7b in the Supporting Information, the CNT/S composite gives a discharge capacity of 940.6 and 507.9 mAh g^−1^ based on sulfur and composite respectively after 30 cycles at a current density of 160 mA g^−1^. The compaction density of CNT/S composite electrodes is ≈0.89 g cm^−3^, and thus yields a volumetric capacity of 452 Ah L^−1^ (Figure 6). It can be calculated that the ratio between the FeS_2_/FeS/S composite and CNT/S composite is increased from 2.0 to 4.7 after taking into account the differences in tap or compaction density.

## 3 Conclusion

In summary, FeS_2_/FeS/S composites employed as cathodes in Li–S batteries have been successfully prepared via a facile, continuous, ball‐milling route. A scalable synthesis, high tap density, high volumetric capacity, and stable cycle performance have been demonstrated. A reversible capacity of 1044.7 mAh g^−1^ and volumetric capacity of 2131.1 Ah L^−1^ after 30 cycles is obtained based on the composites at a current density of 160 mA g^−1^. DFT calculations have been conducted to illustrate the interaction mechanisms between sulfide matrices and polysulfides. Both sulfides possess a strong binding energy in the various forms of Li_2_S*_x_* (*x* = 1,2,4,6,8). This is particularly the case for FeS_2_, which has a strong catalytic effect on the reduction reactions of Li_2_S_6_ and Li_2_S_8_. In addition, we investigated for the first time the effect of the binding direction between polysulfide and host materials (FeS_2_ and FeS in this work). We found experimental evidences that there are weak or negligible interactions when only one Li atom of polysulfides bonds with the sulfur atoms of FeS_2_ and FeS. Moreover, the affinity between FeS_2_, FeS, and polysulfides are also evaluated. All these results in this work demonstrate that a sulfur host is a comprehensive system, which can combine the functions of fast Li^+^/electron transport channels, provide a source of binding sites to anchor polysulfides, and promote electrocatalytic activity for LiPSs. More systematic experiments should be conducted to fully understand the electrochemical kinetics of the sulfur host materials in a Li–S battery. The obtained results open the door for developing high volumetric capacity Li–S batteries when the outstanding FeS_2_/FeS/S composite cathode or its lithiated counterpart is paired with high‐capacity anode materials, such as Si or Sn.

## 4 Experimental Section

*Materials Synthesis and Characterization*: Sodium sulfide nonahydrate (12. 1 g) and sublimed sulfur (4.8 g) were transferred to an agate tank (250 mL), and mixed in a planetary ball mill (QM‐3SP04, Nanjing) at a speed of 350 rpm for 3 h. Afterwards, ferric trichloride hexahydrate (8.2 g) was added, and the ball‐milling continued for a further 3 h at the same speed. The product was then soaked in distilled water for 12 h, before being washed several times with water and ethanol. The black colored FeS_2_/FeS/S composite product was then dried at 50 °C for 12 h under vacuum and stored in a protective atmosphere. The tap density of the composite was tested via a tap‐density meter (JZ‐1, Chengdu Jingxin Powder Analyse Instruments Co. Ltd) with a frequency of 60 times min^−1^ for 30 min. As a comparison, CNT/S composite with a 54.0 wt% sulfur content was also prepared. Typically, sulfur and carbon nanotubes were first mixed via ball milling (300 rpm for 4 h) and then treated at a 155 °C for 6 h to generate the final CNT/S composite.

*Electrochemical Measurements*: The working electrodes were prepared by compressing a mixture of the active materials, acetylene black, and binder (polytetrafluoroethylene, PTFE), in a weight ratio of 70:20:10. The sulfur loading, accounting for the mass ratio of the entire cathode, is 38.9%. The composite cathodes were cut into wafers, of which the active material loading was ≈1.0 mg cm^−2^. The thickness of the cathode is about 90.3 µm and measured by an Exploit Thickness‐gauge (Yiwu Exploit Hardware Co., Ltd). Lithium metal was used as both the counter and reference electrodes. The electrolyte used was Lithium bis(trifluoromethanesulfonyl) imide (2.8 m) dissolved in a mixture of dimethoxyethane and dioxolane in a volume ratio of 1:1. For battery construction, a carbon interlayer, which was prepared by compressing a mixture of active carbon, acetylene black, and PTFE in a weight ratio of 80:10:10, was inserted between the cathode and separator. The area loading mass of this interlayer was 1.0–1.2 mg cm^−2^. LAND‐CT2001A galvanostatic testers were employed to measure the electrochemical capacity and the cycle life of working electrodes at room temperature. The cutoff potentials for charge and discharge were set at 3.0 and 1.5 V (versus Li^+^/Li), respectively. CV experiments were conducted using a CHI 600D, at a scan rate of 0.1 mV s^−1^.

*Assembly of Li_2_S_6_ Symmetric Batteries and Kinetic Study*: FeS/FeS_2_ powders were obtained from FeS_2_/FeS/S composite after washing out sulfur and were loaded onto commercial conductive CP with a loading mass of 0.45 mg cm^−2^. After drying at 60 °C for 24 h, the electrodes was cut into disks with typical diameters of ≈12 mm. 2.5 m Li_2_S_6_ and 0.5 m LiTFSI dissolved in tetraglyme was selected as the electrolyte. For battery assembly, two identical electrodes as cathode and anode, a Celgard 2400 polypropylene membrane as the separator, and 40 µL Li_2_S_6_‐based electrolyte as the active material were used. EIS was performed using potentiostatic mode in the frequency range of 10 KHz to 0.1 Hz with an amplitude of 10 mV. CV was performed at 50 mV s^−1^ within the potential range of −0.8–0.8 V.

*Calculations*: First‐principle calculations based on DFT were carried out by CASTEP.^50^ The electron–electron exchange‐correlation interactions are presented by generalized gradient approximation (GGA) functional.^51^ Ultrasoft pseudopotentials were used with a 330 eV cutoff energy. The supercell models of the sulfide matrix materials were built with atomic slab thicknesses of ≈10 Å in the [110] and [100] directions from Troilite FeS and pyrite FeS_2_, respectively. The selected surface directions are found to be energetically favorable. A vacuum slab with a thickness of 30 Å was deposited on top of the atomic slab. The in‐plane size of each supercell model is ≈10 Å x 21 Å, which is sufficiently large to minimize any interactions with the image systems. All atoms are relaxed during each optimization cycle until the atomic forces acting on each atom are smaller than 0.01 eV Å^−1^ and the energy variation between subsequent iterations falls below 5 × 10^−6^ eV.

The absorbability is evaluated by the binding energy (*E*_b_), which is defined as follows

(1) $$E_{b}=E_{\mathrm{adsorption}}-E_{\mathrm{surface}}-E_{\mathrm{molecule}}$$

where *E*_adsorption_ is the total energy of the FeS*_n_* surface with absorbed Li*_x_*S*_y_*, *E*_surface_ is the total energy of the bare FeS*_n_* surface, and *E*_molecule_ is the total energy of the isolated Li*_x_*S*_y_* molecule.

## Conflict of Interest

The authors declare no conflict of interest.

## Supporting information

Supplementary
