# Supplementary material for: Enhanced Sulfur Transformation by Multifunctional FeS2/FeS/S Composites for High‐Volumetric Capacity Cathodes in Lithium–Sulfur Batteries
Source: Adv Sci (Weinh). 2019 Feb 10;6(6):1800815. doi: 10.1002/advs.201800815 (PMC6425436; doi:10.1002/advs.201800815)
Supplement: Supplementary file 1 — Supplementary [file ADVS-6-1800815-s001.pdf]

Copyright WILEY-VCH Verlag GmbH & Co. KGaA, 69469 Weinheim, Germany, 2019.

ADVANCED SCIENCE
Open Access

Supporting Information

for *Adv. Sci.,* DOI: 10.1002/advs.201800815

Enhanced Sulfur Transformation by Multifunctional FeS_2/FeS/S Composites for High-Volumetric Capacity Cathodes in Lithium–Sulfur Batteries

Kai Xi, Deqing He, Chris Harris, Yuankun Wang, Chao Lai, Huanglong Li,* Paul R. Coxon, Shujiang Ding, Chao Wang,* and Ramachandran Vasant Kumar*

Copyright WILEY-VCH Verlag GmbH & Co. KGaA, 69469 Weinheim, Germany, 2016.

Supporting Information

Enhanced Sulfur Transformation by Multifunctional FeS_2/FeS/S Composites for High-Volumetric Capacity Cathodes in Lithium-Sulfur Batteries

Kai Xi,[‡] Deqing He,[‡] Chris Harris, Yuankun Wang, Chao Lai,[*] Huanglong Li,[*] Paul R. Coxon, Shujiang Ding, Chao Wang,[*] R. Vasant Kumar

Dr. K. Xi, Dr. C. Harris, Dr. P.R. Coxon, Prof. R.V. Kumar
Department of Materials Science and Metallurgy, University of Cambridge, Cambridge CB3 0FS, United Kingdom
Dr. D. He, Prof. C. Lai, Prof. C. Wang
School of Chemistry and Materials Chemistry, Jiangsu Normal University, Xuzhou, Jiangsu 221116, PR China.
E-mail: laichao@jsnu.edu.cn; wangc@jsnu.edu.cn
Dr. Y. Wang, Prof. S. Ding
Department of Applied Chemistry, School of Science, MOE Key Laboratory for Nonequilibrium Synthesis and Modulation of Condensed Matter, State Key Laboratory for Mechanical Behavior of Materials, State Key Laboratory of Electrical Insulation and Power Equipment, Xi'an Jiaotong University, Xi'an 710049, People's Republic of China.
Dr. H. Li
Department of Precision Instrument, Tsinghua University, Beijing, China
E-mail: li_huanglong@tsinghua.edu.cn

Keywords: Lithium-sulfur batteries; FeS_2/FeS/S composites; Volumetric energy density; DFT calculations; Catalytic effect.

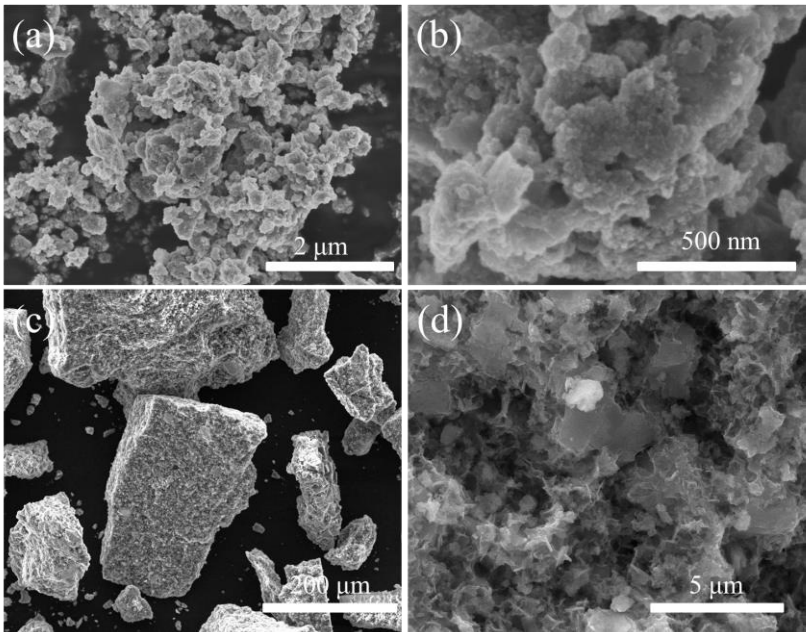

Figure S1. (a,b) SEM images of FeS_2/FeS/S composites after washing with trichloromethane (0.1g composite in 100 mL solvent); (c,d) SEM images of the ball-milled sample without washing.

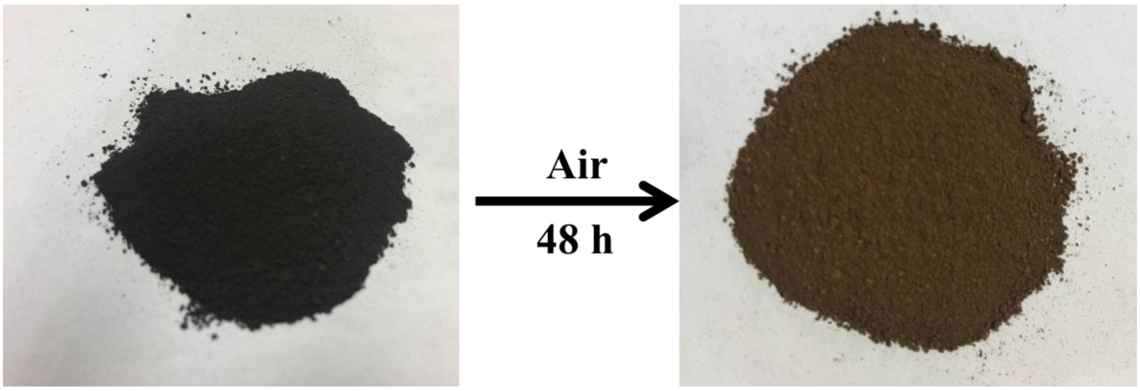

Figure S2. Colour comparisions of FeS_2/FeS/S composites before and after air exposure for 48 h.

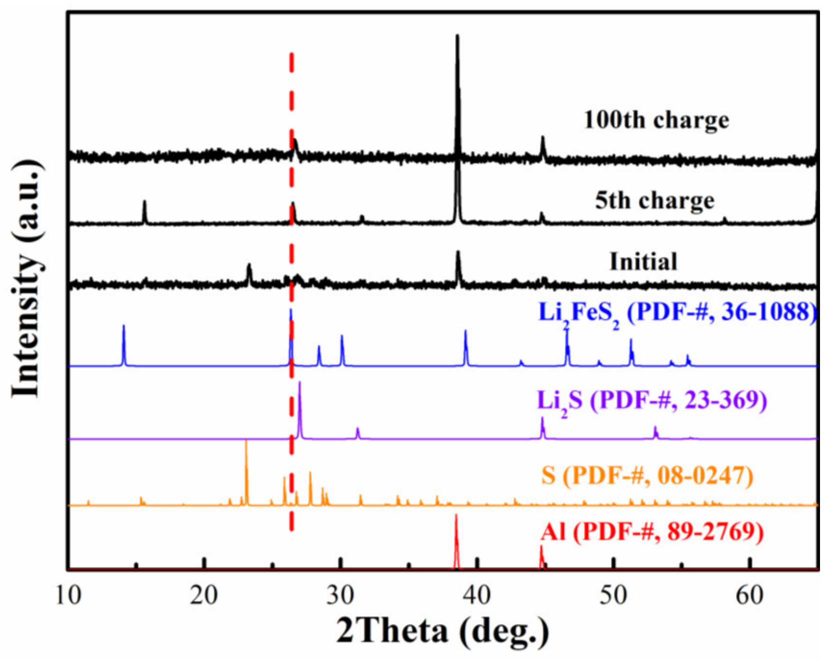

Figure S3. XRD spectra of FeS_2/FeS/S composites electrodes after various cycling times.

Table S1. Comparisons of the volumetric capacity of FeS_2/FeS/S composites and reported sulfur-based cathodes.

Materials	Volumetric capacity ($Ah\cdot L^{-1}$)	Ref
FeS_2/FeS/**S**	1491.7 (30cycle, 0.1C) 746.9 (200cycle, 1.0C) based on electrode	**This work**
rGO–VS_2/S	1182.1 (100cycle, 0.1C) based on electrode	Advanced Energy Materials, 2017: 1702337
Aligned CNT/S	1116 (5cycle, 0.1C) based on electrode	Nano Energy, 2014, 4(2): 65-72
MNCS/CNT-S	1140 (200cycle, 1.68 $mA\cdot cm^{-2}$) based on sulfur(56% in electrode)	Angewandte Chemie, 2015, 54(14): 4325-4329
PSC-CNT-S	800 (80cycle, 0.84$mA\cdot cm^{-2}$) based on sulfur	Acs Applied Materials & Interfaces, 2013, 5(21): 11355-11362
ρPAN-KB/S	576 (100cycle, 0.5C) based on sulfur	Journal of Power Sources, 2016, 302: 70–78
Nafion-coated FGSS	870 (1cycle, 0.1C) based on electrode	Physical Chemistry Chemical Physics Pccp, 2011, 13(17): 7660-7665
GGS	Below 450 (50cycle, 0.1C) based on electrode	Rsc Advances, 2015, 5(59): 47621-47627

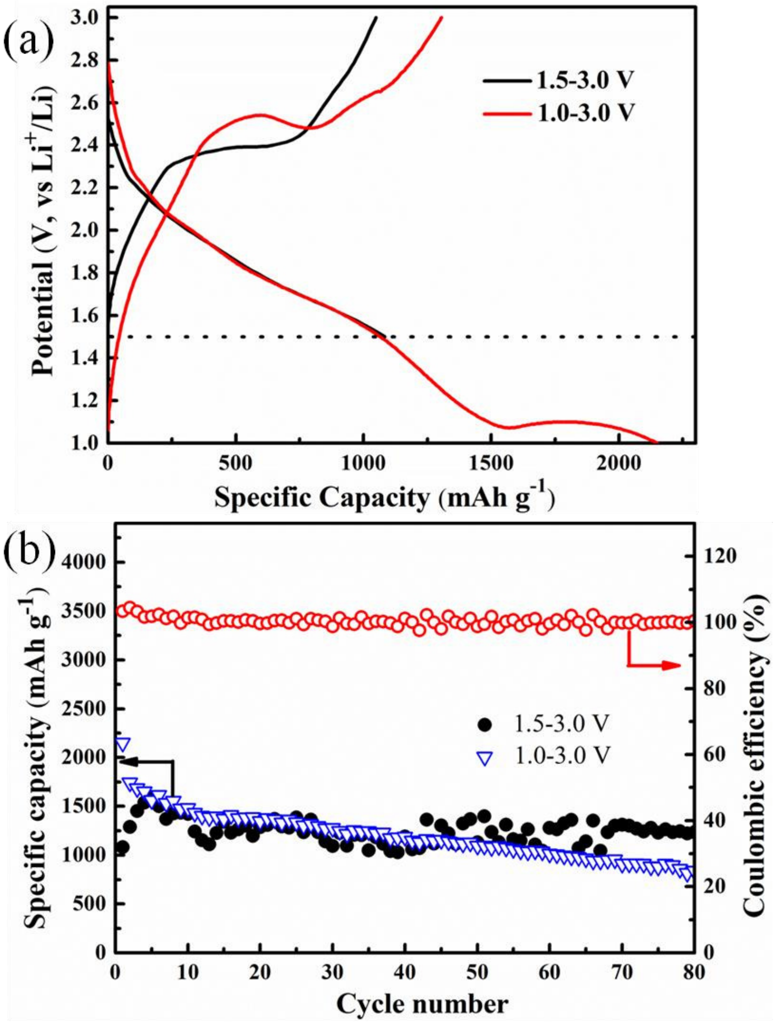

Figure S4. Discharge-charge curves (a) and cycle performance of FeS_2/FeS/S composites at a current density of 300 mA g^{-1} at various cut-off potentials.

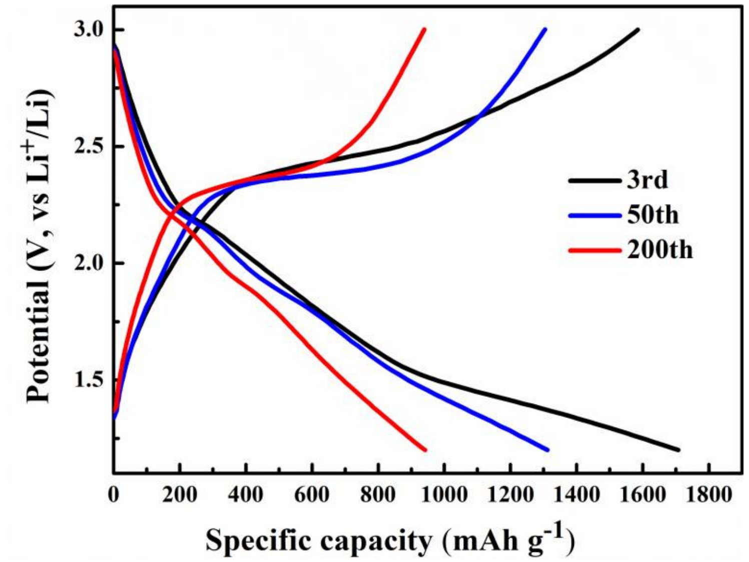

Figure S5. Discharge-charge curves of FeS_2/FeS/S composites at a current density of 1600 mA g^{-1}.

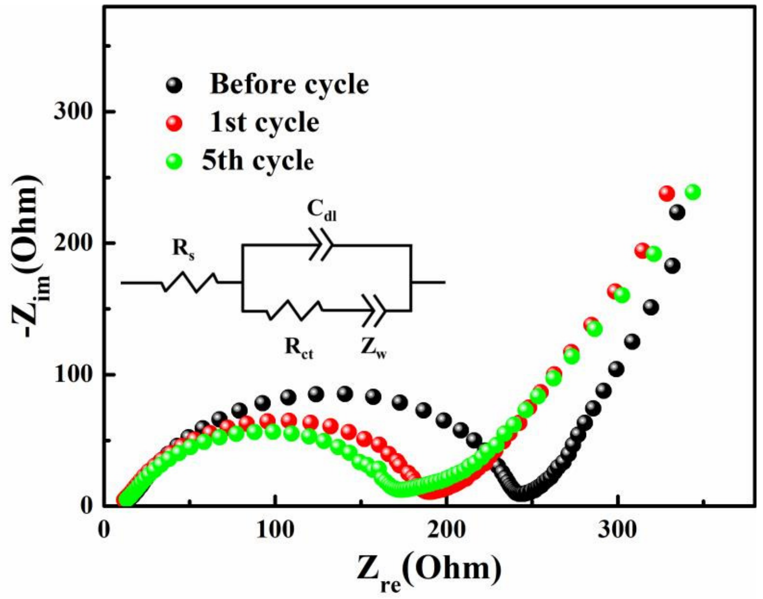

Figure S6. Electrochemical impedance spectroscopy of FeS_2/FeS/S composites.

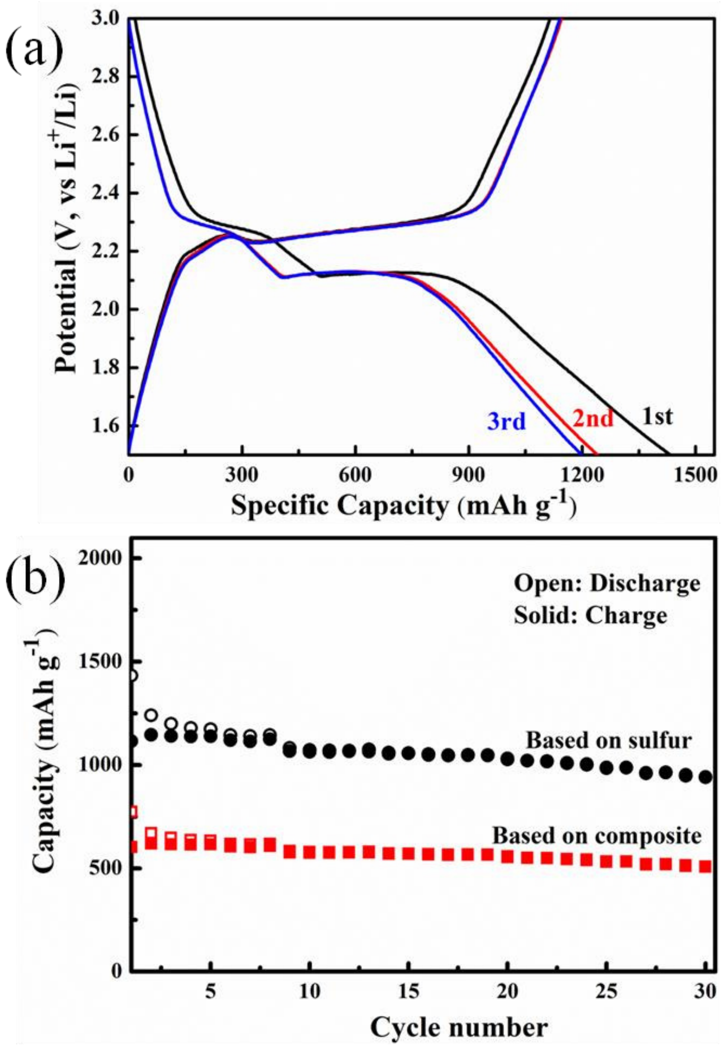

Figure S7. Discharge-charge curves (a) and cycle performance of conventional S/CNT composites at a current density of 160 mA g^{-1}.

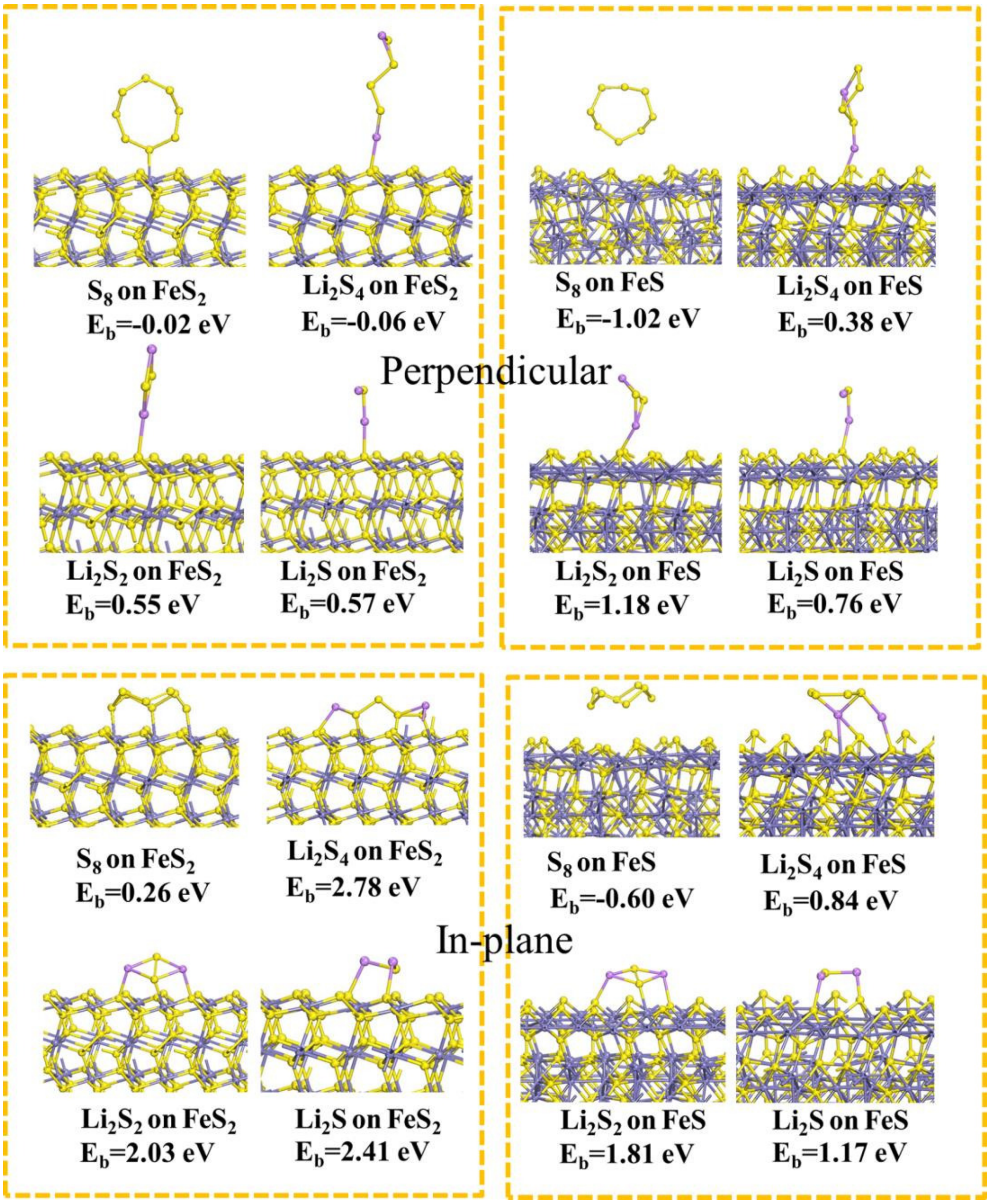

Figure S8. Optimized geometries of S_8, Li_2S_4, Li_2S_2 and Li_2S on FeS_2 and FeS surfaces.

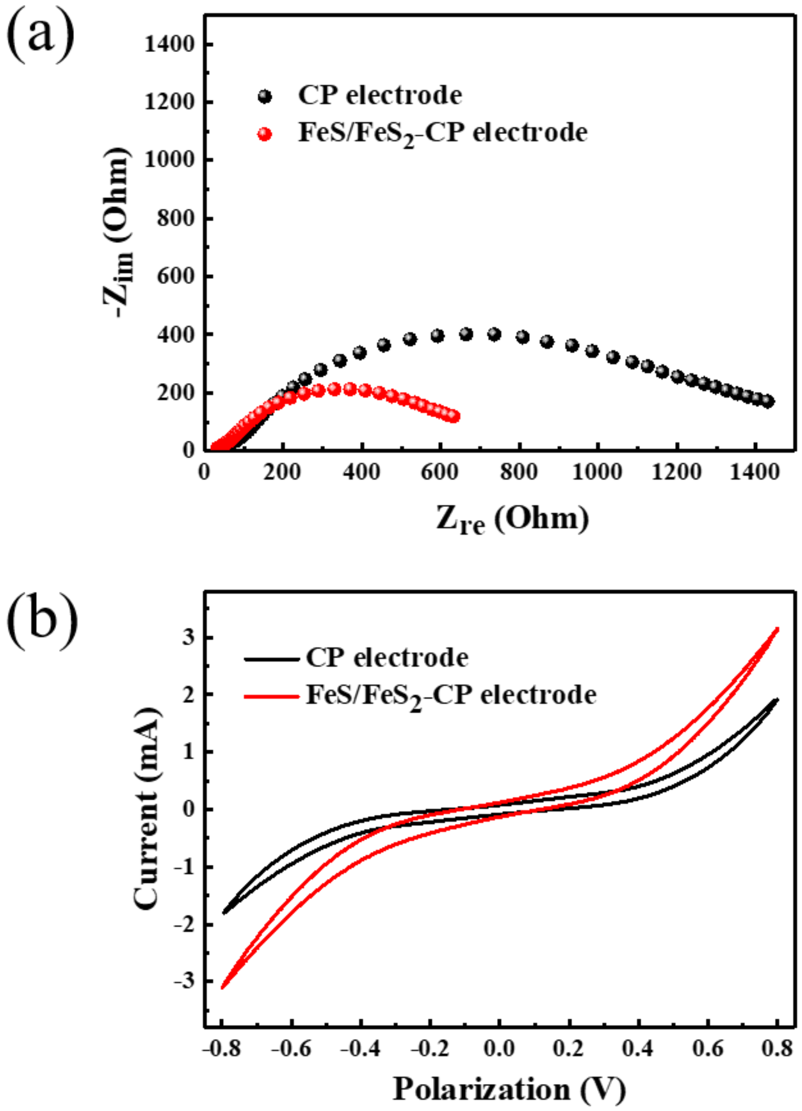

Figure S9. Electrochemical impedance spectroscopy (a) and CVs (b) of CP electrode with or without FeS/FeS_2.
